# Large *T*_g_ Shift in Hybrid Bragg Stacks through Interfacial Slowdown

**DOI:** 10.1021/acs.macromol.0c02818

**Published:** 2021-02-19

**Authors:** Konrad Rolle, Theresa Schilling, Fabian Westermeier, Sudatta Das, Josef Breu, George Fytas

**Affiliations:** † Max-Planck-Institute of Polymer Research, Ackermannweg 10, Mainz 55128, Germany; ‡ Department of Chemistry and Bavarian Polymer Institute, University of Bayreuth, Universitätsstr. 30, Bayreuth 95440, Germany; § Deutsches Elektronen Synchrotron DESY, Notkestr. 85, Hamburg D-22607, Germany

## Abstract

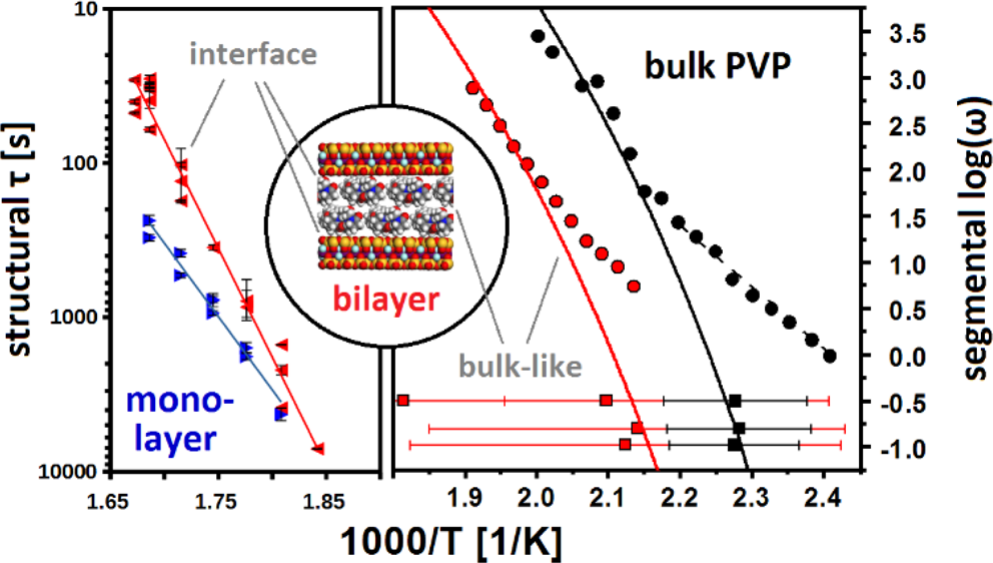

Studies of glass transition under confinement frequently employ supported polymer thin films, which are known to exhibit different transition temperature *T*_g_ close to and far from the interface. Various techniques can selectively probe interfaces, however, often at the expense of sample designs very specific to a single experiment. Here, we show how to translate results on confined thin film *T*_g_ to a “nacre-mimetic” clay/polymer Bragg stack, where periodicity allows to limit and tune the number of polymer layers to either one or two. Exceptional lattice coherence multiplies signal manifold, allowing for interface studies with both standard *T*_g_ and broadband dynamic measurements. For the monolayer, we not only observe a dramatic increase in *T*_g_ (∼ 100 K) but also use X-ray photon correlation spectroscopy (XPCS) to probe platelet dynamics, originating from interfacial slowdown. This is confirmed from the bilayer, which comprises both “bulk-like” and clay/polymer interface contributions, as manifested in two distinct *T*_g_ processes. Because the platelet dynamics of monolayers and bilayers are similar, while the segmental dynamics of the latter are found to be much faster, we conclude that XPCS is sensitive to the clay/polymer interface. Thus, large *T*_g_ shifts can be engineered and studied once lattice spacing approaches interfacial layer dimensions.

## Introduction

Confinement studies on the glass transition temperature *T*_g_(1,2) frequently employ polymer thin films examined first in supported^3^ and later in free-standing^4^ geometries. Because of the susceptibility of *T*_g_ not only to film thickness but also to interfacial conditions, a spatially inhomogeneous *T*_g_ distribution was surmised^3,5^ and finally probed directly^6^ by placing fluorescent markers at different depths inside the sample. This line of investigation continues with efforts to increase spatial resolution, however, struggling with signal strength concomitantly decreasing with thickness. Sample design can address this problem but typically at the expense of having to cater to a specific experimental technique, while barring any multi-pronged characterization approach. The latter, however, is often desirable because complementary to *T*_g_, dynamic data tend to yield new insights. In particular, for broadband investigation of dynamics, technique choice narrows down to dielectric spectroscopy (DS) and photon correlation spectroscopy (PCS), both of which require specific adaptations to probe thin films and their interfaces. For PCS, poor signal from dynamic light scattering^7,8^ warrants its extension to the X-ray domain (XPCS) for interface studies.^9^ For DS, supported (but not free-standing^10^) ultrathin film studies were enabled early^11^ by the DS capacitor geometry; however, spatially resolved (labeling-type) studies are reported much later, and they achieved only 15 nm resolution.^12^ Clearly, a narrow choice of techniques combined with a challenging object of study creates an impasse to be resolved.

Regarding the study of dynamics under extreme confinement, a conceptually straightforward strategy to boost signal at least from supported thin films is to place several layers separated by confining spacers on top of each other. This approach was somewhat prominent in early thin film confinement studies and allows achieving thermal mass sufficient for standard differential scanning calorimetry (DSC) experiments.^13^ In particular, when applying analytical methods that require labeling, it however turned out to be challenging to reproduce the labeling of each individual thin film with sufficient precision in such a thin film stack. Consequently, the approach has not gained much popularity as a universal strategy. However, on the one hand, advanced nanofabrication techniques allow for improved reproducibility between successive polymer layers. On the other hand, the need for alternative experimental methods has become more acute because confinement studies have been focusing on dimensions where measurement becomes ever more challenging. Indeed, particularly interesting is the molecular monolayer and few-layer domain, where a high-viscosity region (often termed “irreversibly adsorbed layer” or “Guiselin brush”)^9,14^ dominates, and relative interface contribution is most important. In order to address this regime, our study will be based on a stacked thin film system, where the number of single polymer layers sandwiched between two (hydrophilic) walls is precisely tunable. The monolayer case is interesting for obtaining the highest *T*_g_ modification respective to bulk, whereas examining higher layer numbers is useful for comparison and deeper understanding. Indeed, by balancing contributions arising from bulk-like and interface regions, an instance of the molecular multilayer case will allow us to confirm two distinct *T*_g_ values from a standard DSC measurement. Similar observations have been made (using other methods) for free surfaces^15^ and buried interfaces^16^ but without the corroborative broadband dynamic data presented here. Thus, our study uses a “stacked thin film” approach^13^ based on a much more reproducible fabrication technique to present the first evidence for a “double *T*_g_” situation from conventional DSC measurements.

**Figure 1:**
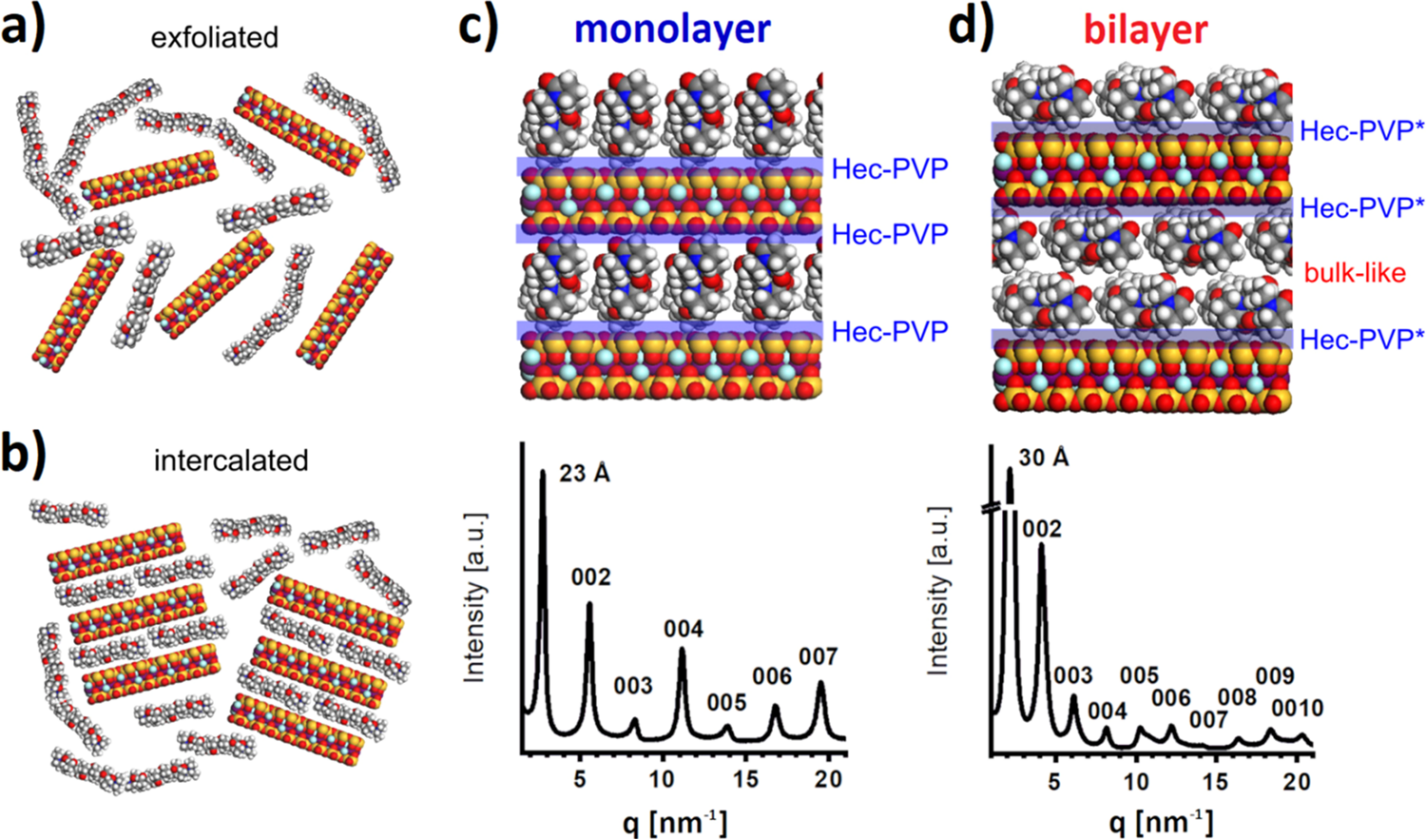
Types of interfaces of various composites and hybrid materials: (a) exfoliated, isotropic and (b) intercalated nanocomposite, (c) and (d) hybrid Bragg stack structure and X-ray diffraction (XRD) pattern for the monolayer (c) and bilayer (d) samples. Top: schematics of the hybrid Bragg stack structure, comprising a single region highlighted in blue (interfacial Hec-PVP) for the monolayer case and two different regions highlighted in red (bulk-like) and blue (interfacial Hec-PVP*) for the bilayer case. To focus on this PVP/hectorite interface, PVP is drawn with space-filling models. For simplicity, ordered arrays of stretched PVP chains in cross-section are shown, while experimental information on chain conformation is not available. Bottom: XRD pattern with a rational series of 00l reflections, showing the *d*-spacings of 23 and 30 Å for monolayer and bilayer cases, respectively (Table 1).

With the advent of various ultrathin filler materials, like nanoclays^17^ or graphene oxide,^18^ nanocomposites became available offering a high specific interface area between nanofillers and polymers. If individual nanosheets can be preserved during compounding, for example, by solution blending, the specific interface area increases with the filler content (Figure 1a). Two confined populations of polymer strands, however, coexist: one at the interface and the other more bulk-like. With small nanofillers and a low filler content, the orientation of nanosheets is random, and the nanocomposite is isotropic (Figure 1a). Τhe distances to the interface range broadly from subnanometer, in wedge-like arrangements, to more bulk-like domains. For some nanocomposites driven by thermodynamics, partial phase segregation is observed. Instead of individual nanosheets, an intercalated hybrid phase is dispersed in the polymer matrix (Figure 1b). With respect to studying dynamics under extreme confinement, this represents an additional complication as two types of nanofiller/polymer interfaces, an external and an intercalated one, need to be taken into account. In addition, the retarded phase segregation kinetics create problems with reproducibility.^19^ For such a “nacre-mimetic” biphasic hybrid system (poly(vinyl alcohol)/montmorillonite), it has already been demonstrated that *T*_g_ is dependent on the hydration level.^20^ For a poly(ethylene oxide)/fluorohectorite system, the immobilized (intercalated) and mobile polymer populations (segregated) were quantified, and the confined polymer dynamics were followed by nuclear magnetic resonance.^21^ For such partial phase-segregated nanocomposite systems, the segregated polymer phase may be “extincted” by applying a nanofiller/polymer ratio corresponding exactly to one of the intercalated domains (Figure 1c,d). This will yield a single-phase material: nanothin polymer layers of well-defined thickness strictly alternating with nanosheets (Table 1 and Figure S3, for a detailed evaluation of the X-ray data). For this one-dimensional (1D) crystal (“Bragg stack”), the height of the gallery between the walls is in the range of the diameter of polymer chains. Because the cross-section of polymer chains is elliptical, like for polyvinylpyrrolidone (PVP, Figure 1), the diffraction data are moreover conclusive for the orientation of the polymer chains relative to the hectorite nanosheets. Consequently, the well-defined periodicity assures that a fixed and finite integral number of polymer layers is intercalated for a given hybrid Bragg stack. To the best of our knowledge, only a single nanoclay/polymer combination has been identified, which comprises a synthetic clay mineral named fluorohectorite and PVP, where hybrid Bragg stacks were observed with a monolayer and a bilayer of PVP (Figure 1c vs d), thereby offering the tunability set out above as a requirement for the present study. Furthermore, the heights of the PVP slabs (Table 1) are much lower than the established *R*_g_ ∼ 13 nm of PVP (40 kg/mol),^22^ preventing the formation of coils in the confined space. The PVP chains with 360 monomers are about 90 nm long (contour length), while the average diameter of the confining nanoplatelets is almost four times that length (340 nm, Figure S1). The confinement is, however, genuinely two-dimensional, so that loops are always possible, but chain entanglements are not allowed in the monolayer case. For the double layer, chains are expected to cross from top to bottom, allowing for chain interpenetration.

**Table 1:** Composition and Polymer Layer Thickness in Two Hec-PVP Bragg Stacks at 0 % RH

sample	nominal Hec:PVP ratio [vol %]	PVP content^a^ [wt %]	PVP content^b^ [vol %]	nominal gallery height [nm]	observed gallery height [nm]	CV^c^ [%]	FWHM^c^ [°2θ]
monolayer	40:60	38	58	1.3	1.3	0.9	0.4–0.7
bilayer	31:69	49	68	2.0	2.0	0.7	0.5–0.7

a Determined by TGA (Figure S2).

b Recalculated from vol % PVP assuming bulk density.

c CV (coefficient of variation); and FWHM (full widths at half maxima) obtained and calculated from the XRD patterns.

Recently,^23^ this material was characterized with a focus on its high thermoelastic anisotropy, where XRD data (similar to Figure 1c,d bottom) were published as well. Also, PVP is a hygroscopic polymer^24^ with a rather high *T*_g_ value (445 K) in the dry state. At these elevated temperatures, increased direct current (DC) conductivity of the material has previously been seen to be a challenge for DS, with no clear observation of the α-, but only β- and αβ-processes claimed.^25^ Along with the strong X-ray signal of the Bragg stack, particularly around the first-order (001) diffraction peak, this suggests the use of XPCS for dynamic characterization. Indeed, the latter has already been applied to inorganic superlattices^26^ (although without scattering from a diffraction maximum) as well as various colloid dynamics studies on clay platelets in suspension.^27−31^ However, the use of XPCS here is warranted foremost because, as we will argue, it exhibits selective interface sensitivity.

## Results and Discussion

**Figure 2:**
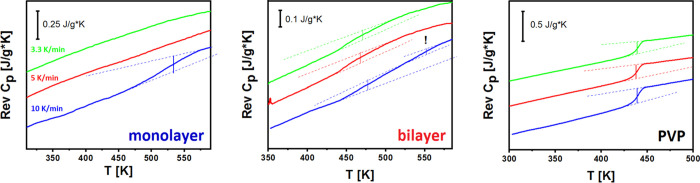
DSC traces for monolayer (left) and bilayer (middle) samples, as well as those for dry PVP (heating rates of 3.3, 5, and 10 K/min from the top to bottom). The scale bar is given per mass of the hybrid Bragg stack.

The monolayer sample (Figure 1c) represents the case of maximum confinement and thus, can be expected to exhibit the highest *T*_g_. Here, at a scan rate of 10 K/min, DSC shows broad glass-rubber transition around *T*_g_ = 537 ± 9 K (bottom trace of Figure 2, left), which is indeed a dramatic^32^ increase with respect to *T*_PVP_ = 437 ± 1 K (Figure 2, right) found for bulk PVP.^25^ A similarly large *T*_g_ increase was recently reported^16^ for poly 2-vinyl pyridine (P2VP) in the vicinity of a glass substrate but can be confirmed here for a true nanocomposite, not a labelled thin film. Alternatively, the large *T*_g_ increase can also result from the slowdown due to the complexation of Na^+^ by PVP because the clay layers carry a permanent negative charge that is compensated by Na^+^ residing in the interlayer space concomitantly with PVP. Thus, the Na^+^–PVP interaction was reflected in small but significant shifts of relevant bands in the infrared spectrum (Figure S4). This is in line with observations on polymer electrolytes: for bulk polypropylene oxide (PPO)/NaCF_3_SO_3_ electrolytes with the monomer/Na ratios of 30:1 and 16:1, the PPO *T*_g_ upshift was about 9 and 19 K, respectively.^33,34^ For the extremely confined monolayer hybrid Bragg stack with a 5:1 monomer/Na^+^ ratio, the observed PVP *T*_g_ increase (∼ 100 K) is, however, clearly much higher than that anticipated (∼ 60 K) based on such a polymer electrolyte effect. Either way, and regardless of quantitative considerations, the general trend of a slowdown is well known to be that for attractive (hydrophilic) interfaces.^35^ The very broad DSC step compared to bulk PVP (Figure 2 right) is indicative of a locally heterogeneous *T*_g_ distribution^20^ and is in agreement with the results on PVP shells from monolayer and bilayer silica core–shell systems.^36^ The latter system failed, however, to show a large *T*_g_ confinement effect, most likely due to the presence of a free surface. In addition to the large *T*_g_ increase, the relaxation strength Δ*C*_p_ of the heat capacity at the glass transition of the monolayer sample is clearly smaller (Δ*C*_p,mono_ = 0.14 J/gK) than that in the bulk PVP (Δ*C*_p,PVP_ = 0.24 J/gK), reflecting the loss of the internal degrees of freedom because of the extreme confinement and complexation of PVP with Na^+^.

**Figure 3:**
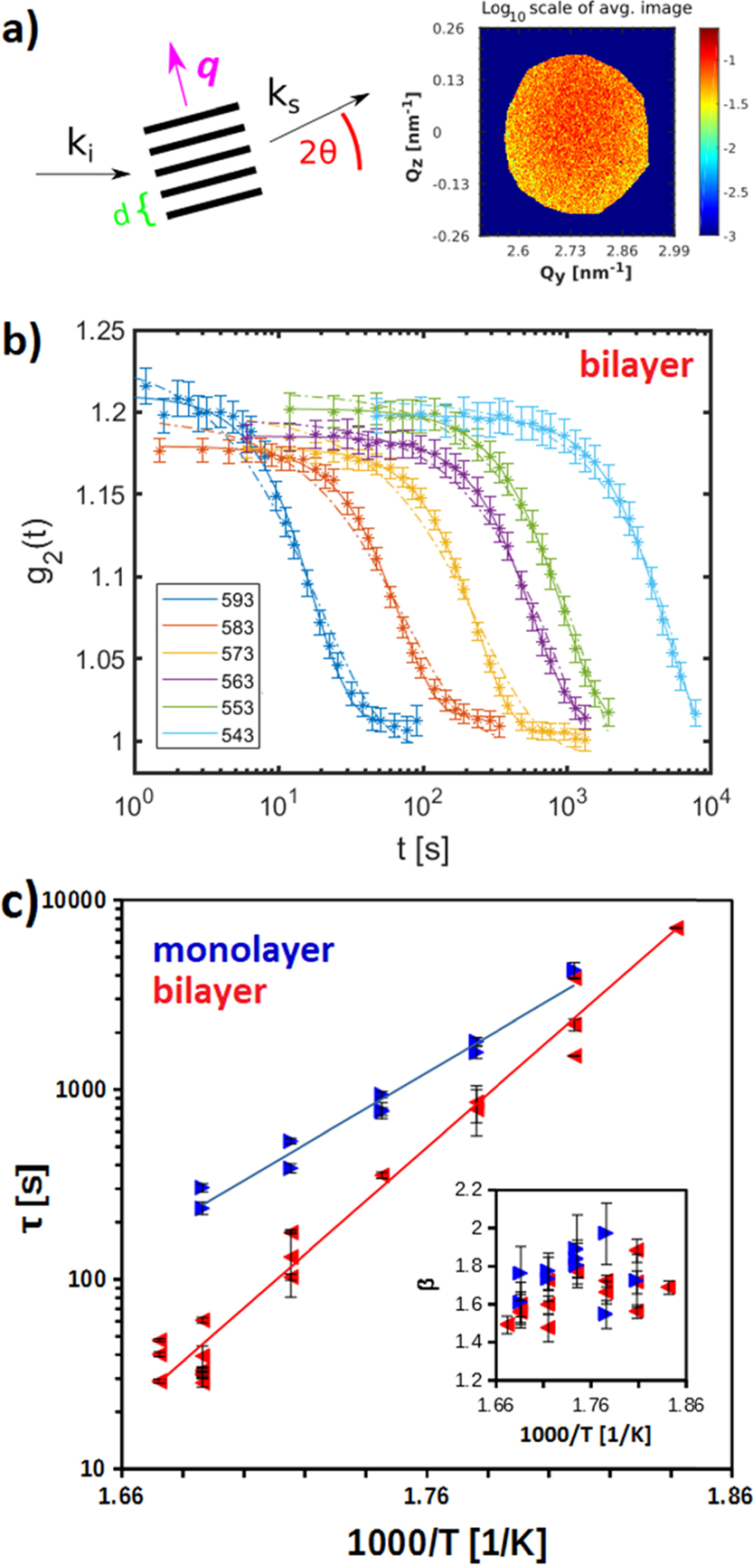
(a) XPCS scattering geometry (left) and typical speckle pattern after masking (right), where ***k*_i_** and ***k*_s_** are the wave vector of the incident and scattered X-ray fields, and ***q**=***k_i_**-**k_s_** defines the scattering wave vector normal to the Bragg stack with periodicity *d* (b) selected XPCS intensity auto-correlation functions of the bilayer sample (monolayer case in Figure S6) at *q*_bi_ = 2.094 nm^–1^ and for different temperatures (decreasing left to right from 593 to 543 K), with solid and dashed lines for compressed and simple exponential fits, respectively (c) XPCS Arrhenius plot for monolayer (▶) and bilayer (◀) samples (inset: the exponent of the compressed exponential fit against temperature); multiple data points for the same temperature can correspond to different repetition rates

The downside of the high *T*_g_ upshift in the monolayer sample is that the resulting high temperature range involved renders DS inapplicable because of increased DC contribution. Conversely, the need for broadband dynamic data is reinforced by the failure of the temperature-modulated (TM) DSC traces to locate *T*_g_ for all but the highest heating rates (Figure 2 left). In view of the broad glass-rubber transition and the very slow dynamics associated with high *T*_g_, the arrest of the dynamic degrees of freedom remains seemingly incomplete at relatively slow rates, resulting in a diffuse cross-over; similar mechanisms seem to underpin the DSC results for the bilayer case, as discussed further below. Accordingly, only structural relaxation times from XPCS are available for characterizing the dynamics in the monolayer sample, which are obtained by correlating intensity speckle patterns (Figure 3a) over time. The experimental intensity correlation functions at different temperatures near *T*_g_ and for fixed *q* (Figure 3b for the bilayer case and Figure S6 for the monolayer) are represented by *g*_2_(*q,t*) *=* α*exp(−2*[Γ(*q*)*t]^β^) *+ A*, with α, Γ(*q*), β, and *A* (∼ 1) being the contrast, relaxation rate, stretching exponent, and baseline, respectively. The shape of the structural relaxation function *S*(*q,t*) *=* [*g*_2_(*q,t*)*-A*]^1/2^ is that of a compressed exponential with β = 1.5 ± 0.2, which is seen to be reassuringly insensitive to temperature variations (inset of Figure 3c). Compressed exponential relaxation is frequently observed in soft^27,28,37,38^ and hard^39−41^ matter systems near *T*_g_, where it is explained as due to “jamming” transitions and their very slow (arrested) collective dynamics. Often, it is accompanied by a hyperdiffusive (or ballistic) behavior (Γ ∼ q). For the dynamics of polymer melts probed by single gold nanoparticle motion, the XPCS relaxation function is a single exponential with a diffusive rate at high temperatures.^42^ With temperature decreasing toward *T*_g_, the relaxation function becomes that of a compressed exponential, with β increasing up to 1.8 close to the polymer *T*_g_, and the dynamics changing from diffusive to hyperdiffusive. Regarding the dynamics of the present hybrid Bragg stack, they bear resemblance to those in the above-mentioned ferroelectric PbTiO_3_/SrTiO_3_ superlattice^26^ and those in anisotropic (peanut-shaped) magnetic colloids aligned normal to an external magnetic field.^43^ In the former, the XPCS results from fluctuations of disordered domains are also described using a compressed exponential function. In the colloidal crystal, XPCS structural relaxation along the magnetic field direction (Bragg direction) is compressed at low *q* with a ballistic rate behavior. Although no clear consensus seems to have emerged in the community on the microscopic mechanism responsible,^40,44^ the compressed exponential is thus not unexpected in the present system.

Relaxation times τ *= 1*/Γ are summarized in the Arrhenius plot of Figure 3c. Platelet dynamics in the monolayer sample are seen to start at temperatures around *T*_g_ as determined by DSC (537 K) where the first XPCS speckle pattern series was obtained at 553 K with ∼ 3 h acquisition time (Figure 3c). Structural relaxation τ(*q*_mono_,*T = T*_g_) is expectedly much longer than segmental relaxation τ_s_ = 20 s, which corresponds to TM-DSC at a heating rate of 10 K/min (τ(*T = T*_g_)/τ_s_ ∼ 540), and it can easily be corroborated by estimating from theory*.* The proper formula for doing so is not known because the *q-*dependence of τ(*q*,*T*) cannot be deduced from observations limited to behavior around the first-order diffraction peak (the sole cue is the compressed exponential, which may point^37,38^ to a *q^–1^* dependence). Such a q-dependent τ(*q*) study around the first-order diffraction peak would be more appropriate for a low *T*_g_ polymer layer like polyethylene oxide. However, for the sake of simplicity, assuming heuristically platelet motion to be diffusive^31,43,45−47^ according to τ *= 1*/(D*q^2^) *=* (16*η*R)/(*k*_B_*T*q^2^) and using the platelet diameter *R* = 340 nm and the Boltzmann constant *k*_B_, we calculate viscosity η ∼ 5*10^7^ Pa*s at 553 K. This value is within the viscosity range reported for bulk PVP with a molecular weight of 44–54 kg/mol near *T*_g_ (433 K).^48^ In this estimation, we ignored the thermodynamic slowdown^31,45−47^ of the collective τ(*q*) at *q*_bi_ at which the structure factor attains its maximum value (Figure 1c). Also, some discrepancy is permissible considering that platelet dynamics can be expected to be slowed down by about an order of magnitude if additional inter-platelet coupling terms are considered.^31,45^

**Figure 4:**
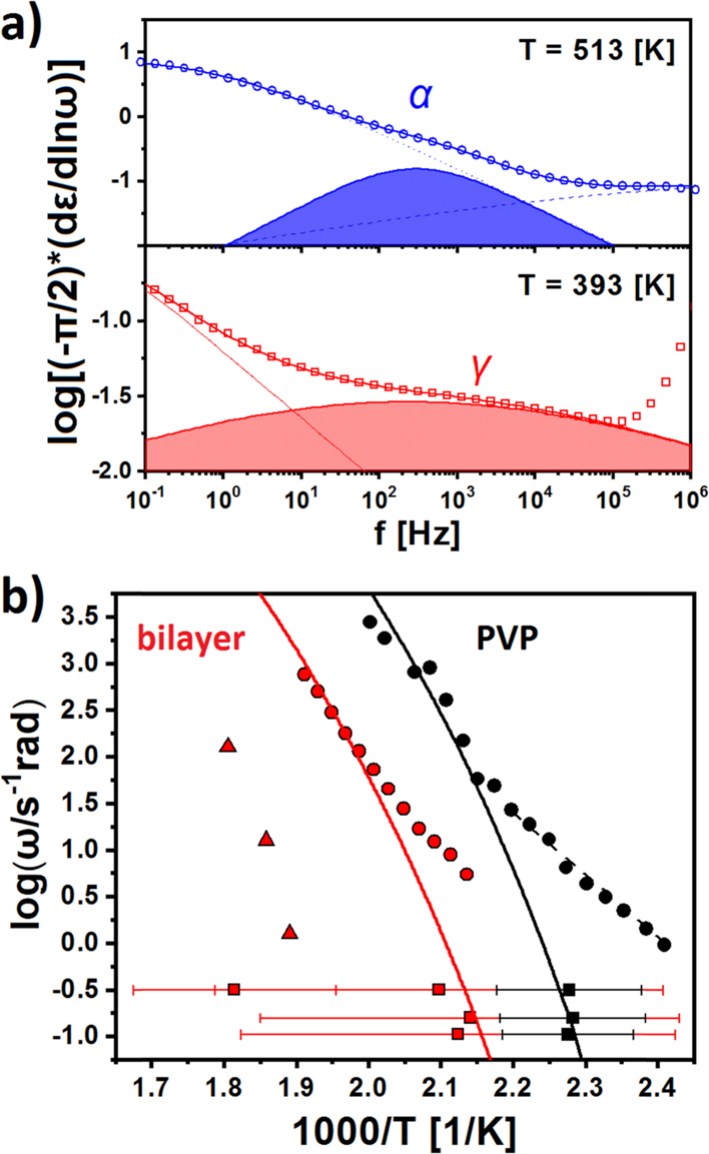
(a) Dielectric spectroscopy (DS) permittivity ε(ω) from the “conduction-free” derivative spectra^52^ (bilayer case) at two temperatures, with electrode-polarization effects (dotted line at low frequency in the top panel) and the Havriliak–Negami representation (shaded areas) for obtaining the segmental (α) and secondary (γ) relaxation process above (513 K) and below (393 K) the lower glass transition temperature *T*_g.l_ = 481 K (dotted line at high frequency in the top panel tail of the γ-process, while solid line in the bottom panel tail of the α-process); the distribution of the relaxation times corresponds to the stretching exponents β = 0.6 and 0.2 for (α) and (γ), respectively (b) Arrhenius plot of the relaxation frequencies 1/τ obtained from DS (●), DSC (■), and DMA (▲) data, for bilayer (composite symbols) and dry PVP (uniform symbols) cases (DS points for PVP from the literature^25^); solid lines are non-Arrhenius Vogel–Fulcher–Tammann (VFT) fits for dry PVP and the process associated with *T*_g,l_ (broken line indicates the PVP β-process), while the DMA points (in default of DS data) can be seen to emulate their behavior for the process associated with the higher transition temperature *T*_g,h_ = 551 K; bars for the relaxation frequencies obtained from DSC (■,□) do not convey *T*_g_ “error bars” but the width of the DSC transition (middle panel of Figure 2)

We surmise that the origin of this large *T*_g_ increase is the slowdown of the segmental dynamics at the adsorbed PVP layer. This is in line with earlier studies,^16^ where two distinct *T*_g_ were observed on the same sample. However, the monolayer shows only a single *T*_g_, probably because its extreme thinness precludes any bulk-like contribution, unlike chain topology in the bilayer Bragg stack (scheme in Figure 1d) with its reduced confinement. Hence, in order to be able to observe both bulk-like and interface dynamics simultaneously, we now turn toward the bilayer sample. The XPCS results are qualitatively quite similar to those obtained for the monolayer case, with the relaxation times only slightly faster (Figure 3c). This small dynamic disparity is indicative of a ∼ 10 K lower *T*_g_, presumably due to reduced confinement. At first glance, a quite different picture emerges from DSC (Figure 2, middle), where all the three traces show a transition that lies around *T*_g,l_ = 481 ± 8 K, which is more than 50 K lower than that in the monolayer with relaxation strength Δ*C*_p,1_ = 0.07 J/gK ∼ Δ*C*_p,mono_/2. However, a closer examination reveals that the trace for the highest heating rate (10 K/min) is actually composed of a double step. The additional transition at *T*_g,h_ = 551 ± 9 K with Δ*C*_p,h_ ∼ Δ*C*_p,1_/3 is up to the experimental uncertainty similar in temperature to the monolayer case (*T*_g_ = 537 ± 9 K as above), which it also resembles in its absence from slower heating rate runs (Figure 2, left). Although this assignment of both *T*_g,l_ and *T*_g,h_ from a single DSC trace might appear overconfident considering the broadness of the step, the high-temperature transition at least is also resolved particularly clearly by dynamic mechanical analysis (DMA, see Figure S7, middle and triangular points in Figure 4b discussed next). In the latter experiment, however, we note that the *T*_g,l_ process is probably masked by a parasitic signal from the clamp. Thus, the stacked thin film approach^13^ provides the first evidence for such a “double *T*_g_” situation from a conventional DSC measurement. A “double *T*_g_” in DSC traces has, however, been reported for plasticized polymers and miscible polymer blends and block copolymers.^49,50^ Moreover the resolution of two *T*_g_s in the bilayer sample is consistent with the size of the correlation length (ξ) associated with the glass transition process.^51^ The computed ξ (∼ 0.8 nm) from the DSC traces of Figure 2, as illustrated in Figure S8, is smaller than the galley heights of the bilayer hybrid stack shown in Table 1.

Because of this discrepancy between the platelet dynamics from XPCS and *T*_g,l_ from DSC, there is interest in examining the segmental PVP dynamics. We can now utilize DS to this end because the PVP composition fraction in the bilayer is higher, and the temperature range suggested by *T*_g,l_ is more amenable to examination by DS than in the monolayer case. Indeed, a weak but sufficient dielectric permittivity signal is in evidence (ε(ω) spectra, as shown in Figure 4a). As described in the Methods section, its analysis was, however, found to benefit from the use of the “conduction-free” derivative spectra (ε” ∼ dε”*/*d*ln*ω from the Kramers–Kronig relationship), where electrode-polarization at low frequency was captured by a power law.^52^ In the fitting procedure, a sum of two Havriliak–Negami (HN) functions (Methods) was used at a given temperature, corresponding to the α/β- and γ-processes. The two processes exhibit different temperature dependences on cooling, as shown in the top and bottom panels of Figure 4a. Below *T*_g,l_, only the high-frequency γ-process is resolved (*T* = 393 K), although the tail of the α-process can be seen at low frequencies (solid line). Above *T*_g,l_, the main process dominates the ε(ω) spectra of the bilayer. At 513 K, the faster γ-process (dotted line at high frequencies) was included in the fitting procedure, and an additional slower process appears (dotted line at low frequencies) that is associated with the ionic conductivity (electrode polarization effects). The relaxation frequency of the γ-process conforms to an Arrhenius temperature dependence (Figure S9). With respect to the α-process, its characteristics change with temperature, from a VFT dependence at higher temperatures (see also bulk PVP, solid black line in Figure 4b) to a process with a weaker temperature dependence on approaching *T*_g,l_. The latter is interpreted as a β-process in the literature^25^ for bulk PVP, although the exact frequency/temperature where the α-process reverts to the β-process is unclear even in this case. However, also considering the data (square points in Figure 4b) from the current TM-DSC study of PVP and the bilayer sample, a clearly seen VFT (as opposed to Arrhenius) dependence for the processes associated with the single *T*_PVP_ in bulk PVP and *T*_g,l_ in the bilayer confirms the assignment of the α-process (note that DSC, in principle, measures the segmental, that is, α dynamics in vitrified systems).

For the VFT fit, the definition is τ_s_*=* τ_o_exp[(*E*/*R*)/(*T-T*_0_)] with the limiting high-temperature time τ_o_ = 10^–12^ s as the fixed parameter, and activation energies *E*/*R* and the ideal glass transition temperature *T*_0_ as the adjustable parameters. From this representation of DS times, *E*/*R* = 4000 ± 200 K and *T*_0_ = 330 ± 10 K. *T*_0_ is low compared to *T*_g,l_ (*T*_g,l_*-T*_0_ = 151 ± 18 K), which results in a near-Arrhenius behavior and indicates a strong glass former. The shape of the DS α-process is characterized by a stretching exponent β = 0.6, suggesting a moderately broad relaxation distribution in contrast to the very broad DSC trace. This finding corroborates the notion that DS probes a single *T*_g,l_. At temperatures below *T*_g,l_, DS reveals an additional very broad secondary relaxation process (γ) shown in Figure 4a bottom with much faster relaxation frequencies exhibiting an Arrhenius temperature dependence (see Figure S9). Finally, the temperature dependence of the segmental dynamics of the bilayer is very similar to that of bulk PVP (*E/R* = 3200 ± 200 K and *T*_0_ = 331 ± 6 K), only shifted upward in temperature by *T*_g,l_*-T*_PVP_ = 44 K.^25^

While comparing segmental dynamics for bilayer and bulk PVP is straightforward, this is less evident for the absolute values of platelet and segmental relaxation times. The much higher temperature of XPCS (Figure 3c) compared to DS times (Figure 4b) for the bilayer, however, presents too large a difference to be explained away by the slower platelet dynamics alone. Fortunately, the comparison becomes clearer if considered in the light of above evidence for a second transition *T*_g,h_. Indeed, the XPCS data for monolayer and bilayer are quite similar, with the Arrhenius fits for both intersecting for τ_mono_(*T*_g_ = 537 K) = 10,770 s at the monolayer *T*_g_, as determined by DSC. Thus, we infer that a *T*_g_ process comparable to that of the monolayer persists in the bilayer as the *T*_g,h_ process, alongside a new *T*_g,l_ process. Hence, the most evident explanation to reconcile the XPCS and DS datasets is to associate them with two different relaxation processes in the polymer, as also reported for polyelectrolytes.^33,34^ However, considering previous results,^15,16^ these relaxation processes are most readily interpreted as pertaining to bulk-like (Figure 1d) and interface regions, with an abrupt transition between both that has been shown in simulations^53^ to occur for certain polymers. The bulk-like process appears alongside the interfacial once the second PVP chain is introduced into the interlayer; however, this neat correspondence between the layer number and *T*_g_ number is likely fortuitous. Indeed, the adsorbed layer can potentially comprise several chains because it is known^54^ to extend over tens of nm in some cases. Thus, the notion of XPCS selectivity to the interface can be deduced from the fact that the faster process associated to the lower *T*_g,l_ is not discernible in the XPCS relaxation functions, and elevates the technique above mere information redundancy with DS. While this selective sensitivity may appear surprising, it is not out of keeping with its previous XPCS studies of viscous adsorbed layers in thin films,^9,55^ in particular, the idea of using markers (e.g. gold particles) to probe local dynamics. Platelets here seem to fulfill a double role of both marker and confinement device for the polymer.

After dwelling on the differences between the XPCS and DS results, considering similarities can help us verify their consistency. While offsets here are difficult to compare quantitatively, this is more obvious for the VFT activation energy *E/R* of structural and segmental relaxations. For the bilayer XPCS times (Figure 3c), a representation using the non-Arrhenius VFT equation, keeping fixed *T*_g,h_*-T*_0_ = 151 K from the DS results, yields *E/R* = 2970 K, while keeping fixed *T*_0_ = 330 K, the activation parameter assumes *E/R* = 3970 K. Thus, the forced VFT fit of XPCS data compares favorably with the value of *E/R* = 4000 ± 200 K from the above discussion of the bilayer DS data (Figure 4b). Alternatively, for the latter, the apparent Arrhenius-energy (*E/R*)/((*1-*(*T*_0_/*T*))^2^ at *T = T*_g,l_ is easily computed to be 40,550 K, which matches the Arrhenius fit of the XPCS bilayer data (Figure 3c) with a value of 32,500 ± 2400 K (here, we are comparing slopes at different temperatures, but both in the vicinity of *T*_g_). However, there is unfortunately some inconsistency between the XPCS slopes from the monolayer and bilayer sample, with the former found to be 22,000 ± 2300 K, which leads to their intersecting around *T*_g_, as mentioned above. Such a flattening of the apparent Arrhenius energy is not to be expected from the VFT curve, considering the hypothesis of similar (interfacial) *T*_g_ values, although it is sometimes observed as a confinement effect^56^ on approaching the length scales of the cooperative dynamics.

Apparent Arrhenius energies can also be related to our recently reported^23^ elastic anisotropy of the nanocomposite from Brillouin light scattering (BLS). In the so-called shoving model^57,58^ for the local segmental relaxation time τ_s_ ∼ exp(*G*(*T*)*V*_c_/*RT*), the total Arrhenius activation energy *E = G*(*T*)*V*_c_ (sum of the local cage and collective long-range rearrangements) can be estimated from the shear modulus *G*(*T*) and the characteristic volume *V*_c_. The application of this formula to an anisotropic system, exhibiting both out-of-plane shear moduli *G_⊥_* and in-plane shear moduli *G_||_*, would require direct access to direction-dependent local segmental dynamics. To the best of our knowledge, we are not aware of such experiments. Turning to structural dynamics, anisotropy effects can be addressed by the vector nature of *q*. For the ordered concentrated suspensions of magnetic anisotropic colloidal particles, XPCS revealed different collective dynamics along and perpendicular to the external magnetic field direction.^43^ The former direction is along the assembled colloidal chains, and here, it corresponds to the out-of-plane direction. Normal to the Bragg plane, shear moduli *G_⊥_* ∼ 1 GPa are lower but close to that of the pure polymer (*G*_PVP_ ∼ 2.5 GPa)^59,23^ and nearly identical between both samples, ruling out a change of slope due to anisotropy. Using PVP density ρ = 1200 kg/m^3^, monomer molecular weight *M* = 0.111 kg/mol, *V*_c_ ∼ *M*/ρ, and neglecting any *G*(*T*) temperature dependence (see also Figure S10) or molecular weight effect correction,^58^ the corresponding Arrhenius temperature estimate is 11,100 K, about half of what was found from the XPCS Arrhenius fits but clearly the right order of magnitude. *G_||_* is much higher, that is, 25.1 GPa (bilayer) and 32.9 GPa (monolayer), which one might expect to see reflected in the Arrhenius energies deduced from DS, which measure an isotropic average. In the DS experiment, the field orientation is normal to the Bragg planes, as with XPCS, but this is only expected to influence the observed relaxation times in very special cases, for example, liquid crystals,^60^ but affects only intensity in, for example, crystalline polymers.^61^ However, as we said, the observed agreement between the “isotropic” DS and “anisotropic” XPCS is quite good; hence, we can conclude that mechanical anisotropy does not seem to impact our results in a significant way.

## Conclusions

We have shown how a clay/polymer Bragg stack enabled by advanced and very reproducible molecular-scale self-assembly techniques can be used to conduct studies of glass transition under confinement on polymer thin films. Particularly, for the challenging problem of interfacial dynamics, it had formerly often been necessary to deploy very creative techniques using specifically modified (e.g., fluorescently labeled) samples to do the same. A shift toward fewer experimental methods and more universally accommodating sample designs can be anticipated with an increasing maturity of the discipline, if suitable new model systems can be identified. By virtue of their supporting characterization using a variety of techniques, we believe that we have shown that nacre-mimetics are a strong candidate. For the problem of interfaces, the tunability of the Bragg stack periodicity (and thereby, polymer layer thickness) has allowed us not only to find a composite with a very high (∼ 100 K) *T*_g_ increase but also to access a regime where signal contributions could be balanced in such a way as to give rise to an appearance of two distinct *T*_g_ values from bulk-like and interface regions in the DSC traces. This resembles results reported in previous studies^15,16^ but with two additional broadband dynamic data sets (from DS and XPCS) for each *T*_g_ to strengthen our case. Depending on the priorities, one could now try to improve on the experiment: while nacre-mimetics benefit from self-assembly methods and the achieved *T*_g_ shift will be a hard rival, they are limited to certain polymer/filler combinations. Thus, alternative fabrication methods, such as layer-by-layer deposition, should be explored, which might also offer nearly unlimited tunability of gallery spacing. Among different fillers, graphene, already the subject of simulations in hybrid stack geometries,^62^ might be an attractive substitute for mica by virtue of its thick adsorbed layer,^54,63^ which in turn might facilitate characterization using different techniques. Finally, we also hope that this study will be a bridge between basic and applied research: Indeed, the present system illustrates an application for confinement effects through its large *T*_g_ increase, which could render it a potential alternative to traditional high-temperature thermoplastics, such as polyimides.

## Materials and Methods

Fabrication of the hybrid Bragg stack films started with synthetic sodium fluorohectorite. Because hectorite shows the rare phenomenon of osmotic swelling, as powder it gently delaminates into 1 nm thick nanoplatelets when immersing it into water. The diameter of as-synthesized hectorite nanoplatelets (median 20 μm) was reduced at this stage to 340 nm by ultrasonication (Figure S1) in order to speed up diffusion dynamics sufficiently to reach timescales easily observable in an XPCS experiment. Mixing the hectorite suspension with varying amounts of an aqueous PVP solution, followed by spray coating, 1D crystalline hybrid Bragg stack films were obtained. Two samples with a hectorite content of 40 vol % (60 wt %) and 31 vol % (50 wt %), respectively, were prepared (for details see Figure S1). These correspond to monolayer and bilayer PVP, respectively, as can be shown by XRD. Indeed, for both compositions, a rational 00l series up to the 7^th^ and 10^th^ order was observed with a *d*-spacing of 23 Å for the monolayer and 30 Å for the bilayer (Figure 1c,d bottom). The quality of the 1D crystallinity of the hybrid Bragg stacks films was corroborated by a low coefficient of variation and small full width at half maximum values with the 00l series. The observed *d*-spacings, furthermore, agreed well with the expectations based on the volume ratios applied (for further information see Figure S2). Indeed, PVP is elliptical with van der Waals radii of the shorter and longer principle axis of 1.0 and 1.3 nm, respectively. This allows to correlate the observed *d*-spacings with a monolayer of PVP oriented with the longer principle axis along the stacking direction (*d*_mono_ = 23 Å) and a bilayer of PVP with the shorter principle axis oriented along the stacking direction (*d*_bi_ = 30 Å). Taking the different orientations of the PVP chains in the monolayer and bilayer sample into account, the interface of the hectorite nanoplatelets with the PVP chain varies (indicated by Hec-PVP and Hec-PVP*). Moreover, for the bilayer sample, a second bulk-like region is created (Figure 1d).

For the XPCS experiment, the samples were vacuum-dried at 100 °C for a week to remove any residual water and then transported to the synchrotron in a desiccator. The measurement was performed in the second experimental hutch of the P10 beamline at Petra III (DESY, Hamburg) at a photon energy of 8.7 keV. The X-rays were focused to a spot of < 5 μm and detected using an Eiger X4M detector at approximately 5 m distance. The samples (thin strips of dimensions ∼ 2 × 10 × 0.03 mm) were mounted on a specially designed copper holder placed in reflection geometry under vacuum, while employing temperature control of the sample environment. On the goniometer, we proceeded to set angles of 2 × θ_mono_ = 3.55° and 2 × θ_bi_ = 2.72°, corresponding to lattice spacings of *d*_mono_ = 23 Å and *d*_bi_ = 30 Å (*q*_mono_ = 2.732 nm^–1^ and *q*_bi_ = 2.094 nm^–1^) for the monolayer and bilayer samples, respectively (Figure 1c,d). A rocking scan was then performed to find the first-order diffraction peak. The attenuation (24 dB) and exposure times (50–100 ms) were chosen to minimize radiation damage throughout the ∼ 300 shots that composed a series of speckle patterns; this threshold was established by first checking at lower temperatures for any spurious decorrelation. Then, samples were heated above *T*_g_, and speckle series were acquired. Generally, acquisitions were run with more than one repetition rate per temperature, which also helped to confirm absence of radiation damage. After each series, a fresh spot on the sample was chosen, and lateral displacement of the sample (which presents a rather narrow profile at low angles) with temperature was compensated for by appropriate readjustments. At the end of the experiment, visual inspection did not reveal any change in the sample appearance, so no decomposition seemed to have occurred.

To support the XPCS measurements, only for the bilayer, DS characterization was performed as a function of temperature in the range from 357 to 526 K using a Novocontrol Alpha frequency analyzer. In all cases, the complex dielectric permittivity ε* *=* ε’–iε”, where ε’ is the real part and ε” is the imaginary part, was obtained as a function of frequency *ω* and temperature *T*, that is, ε*(*T*,ω). Importantly, the real and imaginary parts do not cross within the investigated frequency/temperature range (Figure S11), excluding the possibility of the process being due to ionic relaxation. Figure 4a provides fitting examples using the empirical equation of HN:^64^
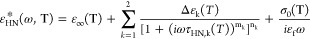
Here, *k* indicates the process under investigation, Δε_k_(*T*) is the relaxation strength, τ_HN,k_ is the relaxation time of the equation, *m*_k_ and *n*_k_ (0 < *m*_k_, *m*_k_*n*_k_ ≤ 1) describe the symmetrical and asymmetrical broadening of the distribution of relaxation times, and ε_∞_ is the dielectric permittivity at the high-frequency limit. The relaxation times at maximum loss τ_max_ have been analytically obtained from τ_HN_ in the HN equation following:
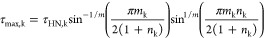


In addition to the measured ε*”*, the derivative of the real part of the dielectric permittivity (dε’/d*ln*ω ∼ (2/π)*ε*”*) was used.^52^ This procedure provides somewhat narrower peaks and suppresses the ionic conductivity (note, however, that the α-relaxation process at high temperatures can be seen in the dielectric loss spectra ε”(ω) as well, as shown in Figure S12). We have employed this approach to locate the frequency maxima of the dynamic processes. Subsequently, fits were performed in the dielectric loss representation with fixed frequency maxima from the derivative approach having free shape parameters. Because of the number of parameters involved, certain fitting criteria were employed. The parameters for the low-temperature γ-process were kept constant to the bulk PVP values. All the dielectric loss curves were fitted several times starting from different initial parameters until they converged to the same final values. Subsequently, the parameters (Figure S13) were further optimized. Minimization was made in Origin Lab 9.0 using the Levenberg–Levenberg–Marquardt algorithm (this iterative procedure combines the Gauss–Newton and the steepest descent method). The respective number of iterations and tolerance were typically 500 and 10^–15^, respectively.

The glass transition of the dried monolayer, bilayer, and PVP samples was recorded by modulated DSC measurements with an amplitude of 1 K and for oscillation periods in the range from 20 to 60 s with the corresponding heating rates from 10 to 3.3 K·min^–1^. The cooling rate (q) was typically 20 K/min, yielding a q to the subsequent heating rate (m) ratio between 2 and 6.^65^ As for the heating rates, we have employed β = (Δ*T*/*nP*)60 s·min^–1^ (Δ*T* is the temperature width of glass transition, *n* is the number of modulation cycles, and *P* is the period of modulation) that ensures 6 cycles within one period for the transition zone in bulk PVP. For the much broader transitions in the hybrid Bragg stacks, the number of cycles is even higher. Finally, for DMA, several (> 10) samples were stacked on top of each other, loaded into a metal clamp and fused by heating to 250 °C. Then, the 30–350 °C range and frequencies between 0.2 and 20 Hz were scanned during the measurements. Argon atmosphere and vacuum-dried samples were used for all of the above-mentioned three methods.

## Supporting Information Available

Describing: sample preparation, characterization, and estimation of the sodium cation to monomer ratio (PDF)

## Abbreviations

TM-DSC: temperature-modulated differential scanning calorimetry
XPCS: X-ray photon correlation spectroscopy
DS: dielectric spectroscopy
DMA: dynamic mechanical analysis
BLS: Brillouin light scattering
HN: Havriliak–Negami
VFT: Vogel–Fulcher–Tammann.
