# Supplementary material for: Large Tg Shift in Hybrid Bragg Stacks through Interfacial Slowdown
Source: Macromolecules. 2021 Feb 19;54(5):2551–60. doi: 10.1021/acs.macromol.0c02818 (PMC8016143; doi:10.1021/acs.macromol.0c02818)
Supplement: Supplementary file 1 — ma0c02818_si_001.pdf [file ma0c02818_si_001.pdf]

Supporting Information

Large T_g Shift in Hybrid Bragg Stacks through Interfacial Slowdown

Konrad Rolle[1]*, Theresa Schilling*[2]*, Fabian Westermeier*[3]*, Sudatta Das*[1]*, Josef Breu*[2]*, George Fytas*[1]*

[1]Max-Planck-Institute of Polymer Research, Ackermannweg 10, 55128 Mainz, Germany

[2]Department of Chemistry and Bavarian Polymer Institute, University of Bayreuth, Universitätsstr. 30, 95440 Bayreuth, Germany

[3]Deutsches Elektronen Synchrotron DESY, D-22607 Hamburg, Germany

*fytas@mpip-mainz.mpg.de

S1. Sample preparation

Materials

The synthetic clay sodium fluorohectorite (Hec, $[Na_{0.5}]^{inter}[Mg_{2.5}Li_{0.5}]^{oct}[Si_4]^{tet}O_{10}F_2$) was synthesized by melt synthesis followed by long-term annealing, according to an already published procedure.[1, 2] The material featured a cation exchange capacity of 1.18 mmol g^{-1}.[1, 2] Upon immersion in deionized water, the pristine material swells osmotically producing a nematic suspension with nanoplatelets of mean diameters of ≈ 20 µm being separated by > 157 nm at 0.2 vol %. To reduce this diameter, a 0.2 vol % suspension of the pristine Hec was sonicated for 15 minutes in an ice bath applying a UIP 1000hd (Hielscher Ultrasonic GmbH, Germany) equipped with a ultrasonic horn BS2d22 and a booster B2-1.2, at 20 kHz with a maximal output power of 1000 W.

The sonicated suspensions were diluted (0.0004 vol %), drop coated on a plasma-treated silicon wafer and sputtered with 10 nm carbon for scanning electron microscopy (SEM) applying a Zeiss Ultra plus (Carl Zeiss AG, Germany) at an operating voltage of 3 kV. *ImageJ* was used to evaluate the average diameter (340 nm) of 125 nanoplatelets (Figure S1)

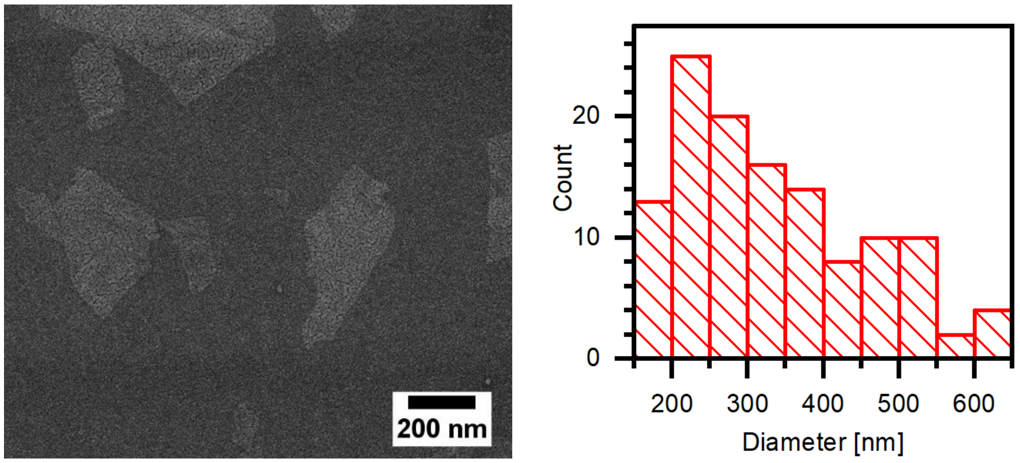

Figure S1. Typical SEM micrograph and histogram of the platelet diameter distribution of sonicated Hec as evaluated by *ImageJ*.

Film preparation

An aqueous Polyvinylpyrrolidone (PVP; Mw = 40.000 g mol^{-1}, Sigma Aldrich) solution (0.8 vol %) was added in appropriate amount to obtain the desired volume ratio of Hec/PVP. To assure homogenization, the suspension was mixed for 1 day in the overhead shaker.

The self-supporting films were prepared by spray coating. The fully automatic spray coating system was equipped with a SATA 4000 LAB HVLP 1.0 mm spray gun (SATA GmbH & Co. KG, Germany). Suspensions were sprayed on a corona-treated polyethylene terephthalate (PET) foil (optimont 501, bleher Folientechnik, Germany). The spraying and nozzle pressure were set constant at values of 2 and 4 bar, respectively. The round per flat fan control was set to 6 with a flow speed of 3

mL s^{-1}. The distance between the spraying gun and the substrate was 17 cm. The thickness of the suspension layer applied in one spraying step is about 2 µm which corresponds to about 20 nm dry film thickness. For drying the suspension layer, the sample is stopped under infrared lamps until evaporation of the solvent is complete. After every spraying cycle, a drying cycle of 90 s with a temperature of 55 °C took place. The spraying/drying cycle is repeated until the desired barrier film thickness of 40 µm is obtained. Afterward, the film was dried at 100 °C for 3 days and peeled off from the PET foil for achieving self-supporting films. In total, we prepared two different samples: Hec40/PVP60 (Monolayer) and Hec31/PVP69 (Bilayer).

These ratios were cross-checked (Figure S2, Table 1) for the dried films by thermogravimetric analysis (TGA), using a Mettler Toledo SDTA851 equipped with the gas control unit TS0800GC1 (Mettler Toledo, USA). Changes in mass observed upon heating in synthetic air up to 900 °C were attributed to the combustion of PVP. (Figure S2).

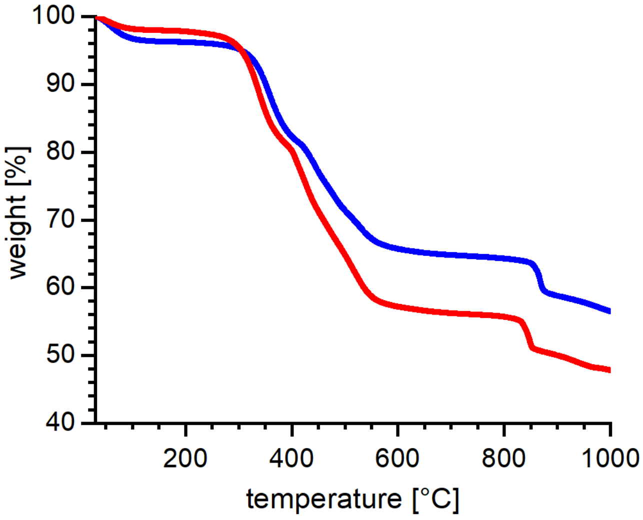

Figure S2. TGA curves of the monolayer (red, Hec40/PVP60) and bilayer (blue, Hec31/PVP69) sample. The weight loss below 150 °C corresponds to adsorbed water.

S2. Characterization of the films

XRD patterns for the films were recorded in Bragg-Brentano-geometry on an Empyrean diffractometer (PANalytical B.V.; the Netherlands) using Cu K_{α} radiation (λ = 1.54187 Å). The self-supporting films were placed on glass slides (Menzel-Gläser; Thermo Scientific). Before the measurements, samples were dried at 100 °C for one week in a vacuum chamber. For both compositions, a rational *00l* series was observed with *d*-spacings of 23 Å and 30 Å for Hec40/PVP60 and H31/PVP69, respectively (Figure 1). The stacking of Hec nanoplatelets and PVP upon drying the nematic suspensions during film preparation was of course turbostratic, meaning that Hec and PVP layers are randomly shifted and rotated relative to each other. Nevertheless, the orientation of PVP chains relative to the Hec nanoplatelets could be solely determined by a detailed analysis and

interpretation of the well-defined periodicity along the stacking direction in combination with the finite dimensions and shape of PVP.

While XRD would be blind for segregated amorphous polymer volumes, partial phase segregation, as observed e.g. for Hec/PEG,[3] can be safely ruled out by the features of the *00l*-series. Any defects in the stacking, any variation of the height of the PVP layer sandwiched between Hec nanoplatelets would according to Mering´s principles[4] instantly and with high sensitivity lead to irrationality and a non-physical varying broadening of *00l* reflections. The XRD patterns observed for both compositions showed, however, a nicely rational *00l*-series as indicated by a low coefficient of variation (CV) and the full widths at half maxima (FWHM) of individual reflections being very similar (Table 1). This proves that the hybrid films are periodically homogenous over long ranges. The sensitivity of FWHM and CV criteria were tested by varying the compositions of the two hybrid films, which will force defects to be incorporated into the periodic domains to adjust the stoichiometry. Even slight deviations from the ideal compositions lead to significant increases in both CV and FWHM of the *001* reflection.

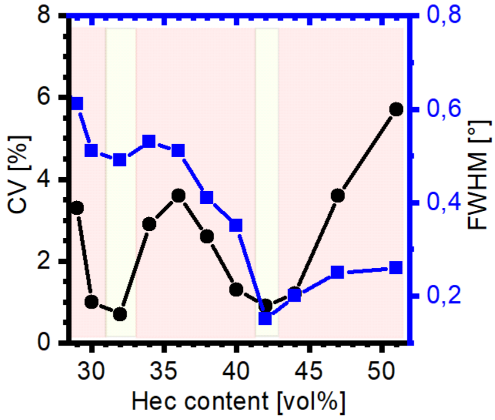

Figure S3. Variation of CV (black circle) and FWHM (blue square) with varying Hec volume content. The minima at Hec32/PVP68 and Hec42/PVP58 confirm the best one-dimensional periodicity.

Next, the *d*-values allowed to calculate the volume fraction of PVP in the periodic domains. Subtracting the known thickness of Hec nanosheets (9.67 Å)[5] from the *d*-spacing of the Hec/PVP film compositions of Hec40/PVP60 and Hec31/PVP69, values that are in close agreement to compositions applied in manufacturing the films were obtained. This close agreement suggests that within experimental error all available PVP was included in the periodic domains and no extra, segregated PVP volumes exist. Finally, the orientation of the PVP chains could be deduced by simply comparing the thickness of the PVP layers as derived by PXRD of films with molecular dimensions. PVP is elliptical with van der Waals radii of the short and long principle axis of 1.0 and 1.3 nm. Since the Hec surface is corrugated allowing PVP to protrude into the rough surface, these dimensions correlate well with a monolayer of PVP oriented with its long principle axis along the stacking direction in Hec40/PVP60 and a bilayer of PVP with its longer principal axis oriented in the plane of the PVP layer for Hec31/PVP69. Since wrong orientations would locally increase the thickness of the PVP

layer by some 3 Å, such defects in the periodicity would be detectable even at low concentration by affecting the rationality of the *00l*-series, which was not the case.

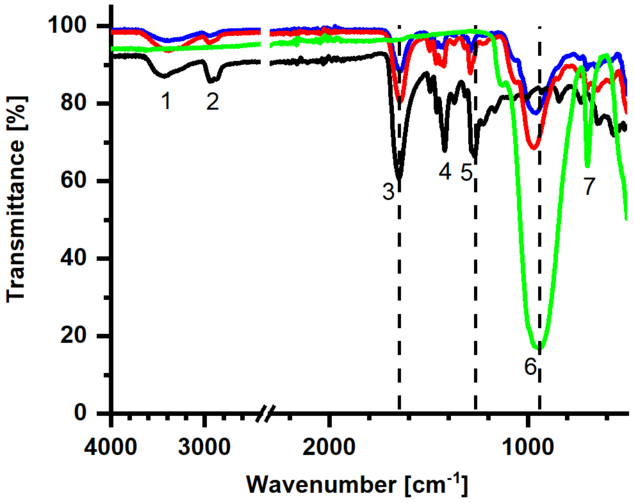

Figure S4. IR spectra of Hec (green), PVP (black), monolayer (blue, Hec40/PVP60) and bilayer (red, Hec31/PVP69) hybrid films.

Infrared (IR) spectroscopy was performed on a JASCO FT/IR-6100 Fourier transform IR spectrometer (JASCO Corporation, Japan) equipped with an attenuated total reflectance (ATR) unit. The infrared spectra of Hec (Figure S4, green) shows two characteristic and dominant bands: 6) the main Si-O stretching vibration at 945 cm^{-1} and 7) the perpendicular Si-O bending vibration at 700 cm^{-1}.[6] The characteristic bands of PVP (Figure S4, black) can be assigned to 1) the O-H stretching vibration of water at 3440 cm^{-1}, 2) the C-H stretching vibration of the polymer backbone (2920 cm^{-1}), 3) the C=O stretching vibration at 1650 cm^{-1} 4) the H-C-H bending vibration (1420 cm^{-1}) and 5) the C-N bending vibration (1267 cm^{-1}).[7] In the Hec/PVP nanocomposites, the C=O stretching band (3) is slightly shifted to lower wavenumbers (1643 cm^{-1}). This red shift arises from the complexation of Na^+ monopoles with partially negative charged oxygen of the carbonyl group (Figure S6a) being accompanied with a decrease in the electron density in the carbonyl bond. This red shift was also observed when PVP was used in the steric stabilization of nanoparticles.[7, 8] In contrast, the C-N bending vibration is shifted to higher wavenumbers from 1267 cm^{-1} to 1291 cm^{-1}. Considering the resonance structure of PVP (Figure S6b), the formation of negatively charged oxygen atom is accompanied by a double bond formed by the lone pair of the nitrogen atom. This double bond increases the electron density and therefore the vibration energy of the C-N bond and results in a blue shift.

Moreover, the main Si-O stretching vibration is also increased from 945 cm^{-1} to 982 cm^{-1} in the Hec/PVP nanocomposites. This might indicate a possible interaction between the oxygen atoms in the tetrahedral sheet of Hec and the partially positive charged nitrogen atoms of PVP.

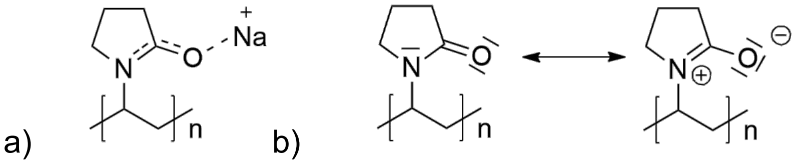

Figure S5: a) Resonance structure of PVP and b) proposed complexation of Na^{+} by PVP.

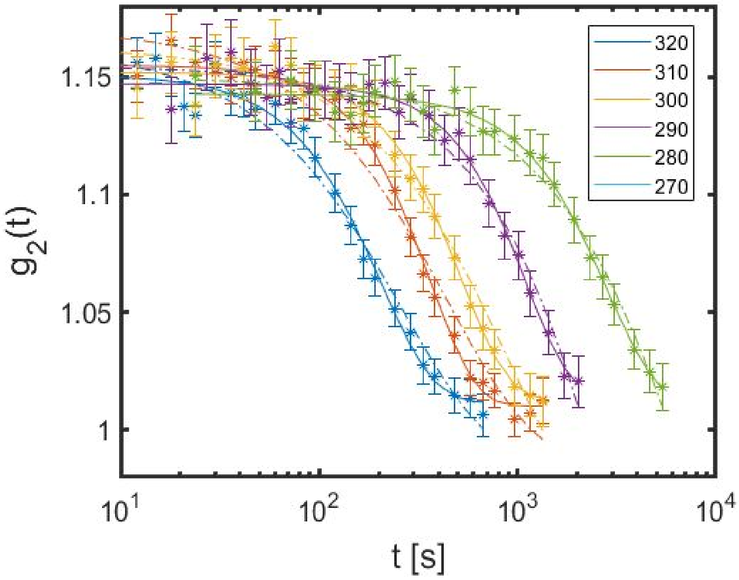

Figure S6. Selected XPCS correlation functions for monolayer sample at q_{mono}=2.732 [nm^{-1}] and different temperatures in [°C] (decreasing left to right)

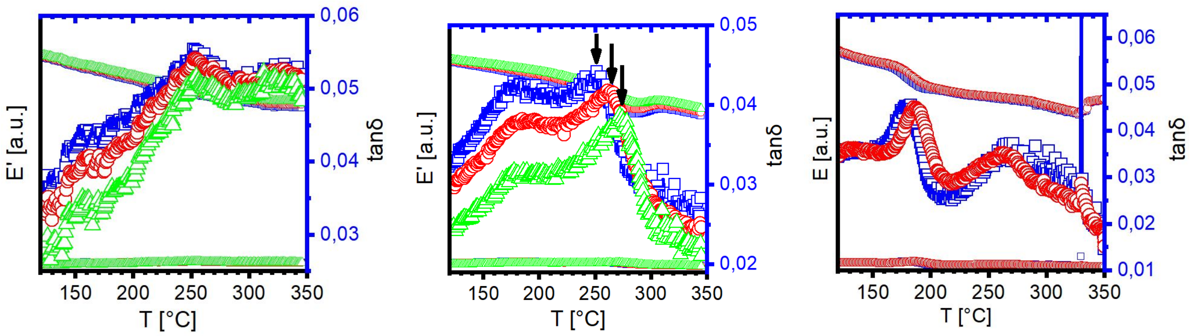

Figure S7. DMA measurements for the monolayer (left, Hec40/PVP60), bilayer (middle, Hec31/PVP69) and PVP (right) at three different frequencies (0.2 Hz – blue square, 2Hz – red circle and 20 Hz – green triangle). Due to the measurement in metal clamps, the unit for the E modulus can only be given in arbitrary units. Also, the very weak E' step is probably due to a parasitic signal from the clamp which could mask the $T_{g,l}$ (=208 ^{0}C) process for the bilayer. Hence, in the main text, we only use data points for the $T_{g,h}$ (=278 ^{0}C), where strong corroboration is obtained through the observed frequency dependence (arrows).

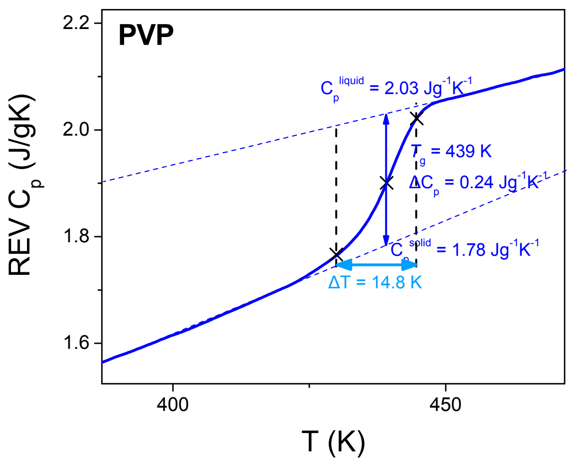

Figure S8. Estimation of the thermal parameters needed to compute the correlation length associated with the glass transition, for example in bulk PVP. The correlation length associated with the segmental (α–) relaxation can be estimated[9] from the step in heat capacity as $\xi_\alpha = \left(\frac{k_B T_g^2 \Delta(1/c_p)}{\rho(\delta T)^2}\right)^{1/3}$ and $\Delta(1/c_p) = \frac{1}{C_p^{Glass}} - \frac{1}{C_p^{Liquid}}$ The procedure along the meaning of the parameters necessary to compute ξ is illustrated in Figure S8 for bulk PVP. The correlation length amount to ~ 0.7 nm and ~ 0.9 nm respectively for the $T_{g,l}$ and $T_{g,h}$. Hence both length scales are smaller than the nominal gallery height (Table 1).

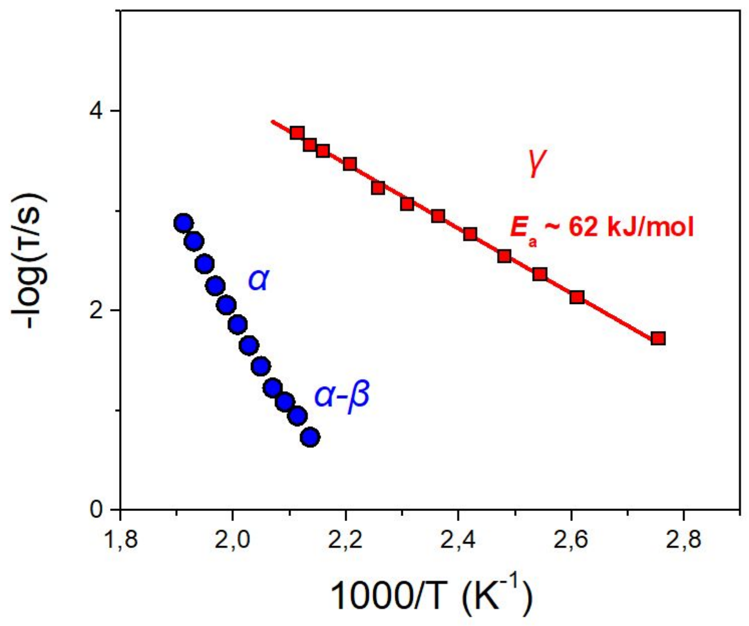

Figure S9. γ-process for bilayer sample from DS, with Arrhenius-fit

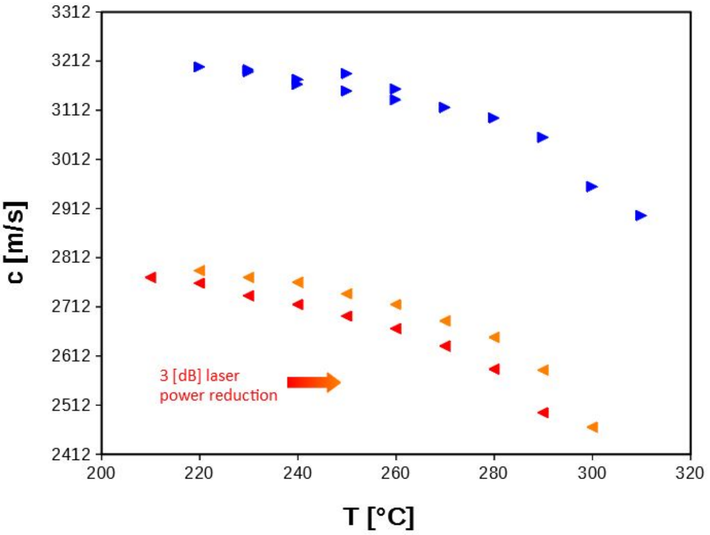

Figure S10. Sound velocity against temperature for vacuum-dried monolayer (▶) and bilayer (◀) samples (from 110 [°] reflection geometry BLS measurement, assuming n_{mono}=1.47 and n_{bi}=1.5). No argon atmosphere was used, but for this T_g investigation technique, heating by the laser (bilayer case datasets) seems to further restrict the already narrow range between T_g and decomposition temperature

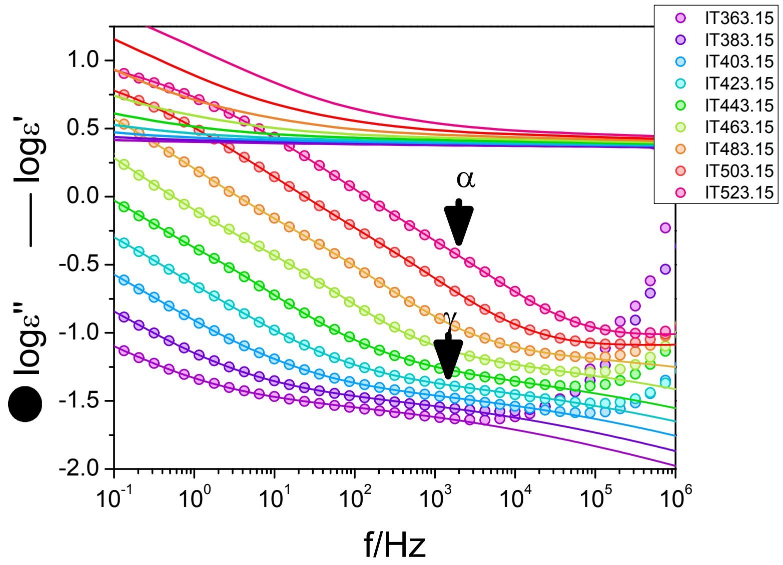

Figure S11. Dielectric permittivity and loss data of the bilayer case for a range of temperatures. The real and imaginary parts do not cross within the shown frequency range.

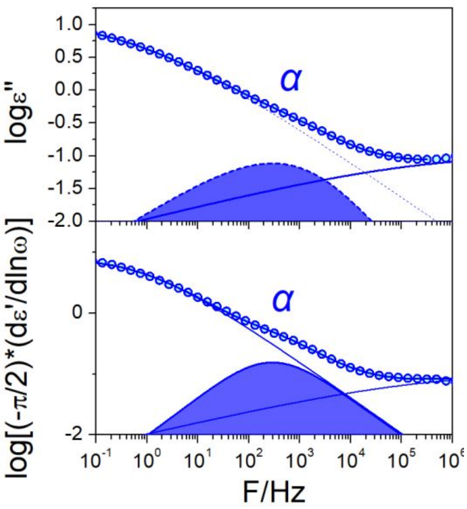

Figure S12. (Top) Dielectric loss data of the bilayer case and (bottom) the first derivative of the dielectric permittivity with respect to frequency at the same temperature (*T*=513.15 K). The process is more evident in the derivative representation.

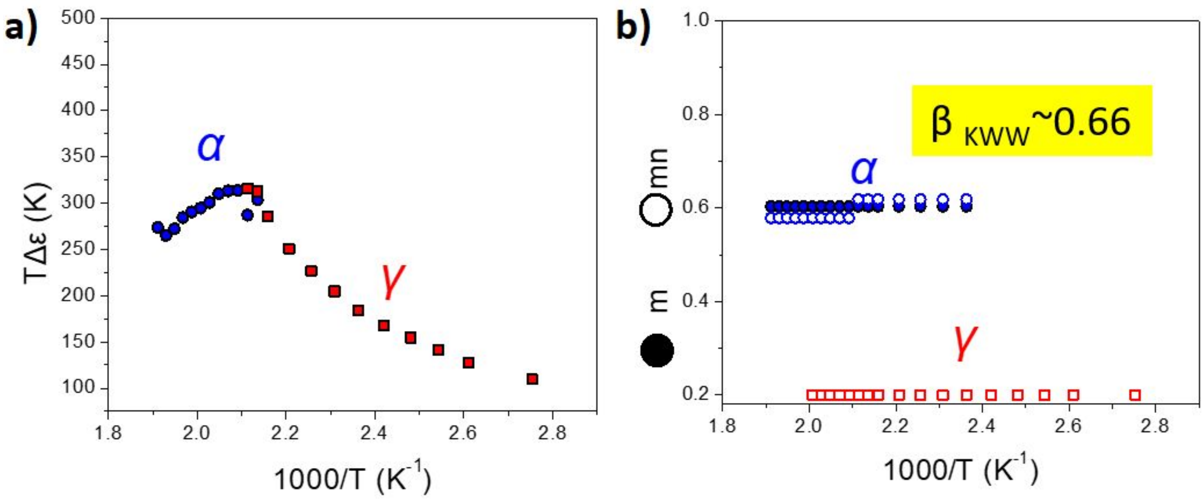

Figure S13. Additional fitting parameters for DS data as in Figure S9, showing a) dielectric relaxation strength Δε and b) symmetric (*m*) and asymmetrical (*n*) broadening of the relaxation times

S3. Estimation of the sodium cation to monomer ratio

1) Sample Hec31/PVP69 → Hec:PVP ratio [wt %] = 50:50

   Assumption: 1 g Hec + 1 g PVP

   Calculation of amount of substance of sodium cation:

$$1g * 1.18\frac{mmol}{g} = \mathbf{1.18\,mmol}$$

   With cation exchange capacity of Na-Hec of 1.18 mmol/g

Calculation of monomer concentration:

$$\frac{1g}{111\frac{g}{mol}} = 0.009\,mol = \mathbf{9\,mmol}$$

Sodium cation to monomer ratio [%] = **12:88**

2) Sample Hec40/PVP60 → Hec:PVP ratio [wt %] = 60:40

Assumption: 1g Hec + 0.67 g PVP

Calculation of sodium cation concentration:

$$1g * 1.18\frac{mmol}{g} = \mathbf{1.18\,mmol}$$

With cation exchange capacity of Na-Hec of 1.18 mmol/g

Calculation of monomer concentration:

$$\frac{0.67g}{111\frac{g}{mol}} = 0.006\,mol = \mathbf{6\,mmol}$$

Sodium cation to monomer ratio [%] = **16:84**

Literature

1. Breu, J.; Seidl, W.; Stoll, A. J.; Lange, K. G.; Probst, T. U., Charge Homogeneity in Synthetic Fluorohectorite. Chem. Mater. 2001, 13 (11), 4213-4220.
2. Stöter, M.; Kunz, D. A.; Schmidt, M.; Hirsemann, D.; Kalo, H.; Putz, B.; Senker, J.; Breu, J., Nanoplatelets of sodium hectorite showing aspect ratios of approximately 20,000 and superior purity. Langmuir 2013, 29 (4), 1280-1285.
3. Habel, C.; Maiz, J.; Olmedo-Martínez, J. L.; López, J. V.; Breu, J.; Müller, A. J., Competition between nucleation and confinement in the crystallization of poly(ethylene glycol)/ large aspect ratio hectorite nanocomposites. Polymer 2020, 122734.
4. Moore, D. M.; Reynolds, R. C.; M., D., X-ray Diffraction and the Identification and Analysis of Clay Minerals. Oxford University Press: Oxford, U.K., 1997.
5. Kalo, H.; Milius, W.; Breu, J., Single crystal structure refinement of one- and two-layer hydrates of sodium fluorohectorite. RSC Adv. 2012, 2 (22), 8452-8459.
6. Madejová, J.; Gates, W. P.; Petit, S., IR Spectra of Clay Minerals. Developments in Clay Science 2017, 8, 107-149.
7. Safo, I. A.; Werheid, M.; Dosche, C.; Oezaslan, M., The role of polyvinylpyrrolidone (PVP) as a capping and structure-directing agent in the formation of Pt nanocubes. Nanoscale Adv. 2019, 1 (8), 3095-3106.
8. Teranishi, T.; Hosoe, M.; Tanaka, T.; Miyake, M., Size Control of Monodispersed Pt Nanoparticles and Their 2D Organization by Electrophoretic Deposition. J. Phys. Chem. B 1999, 103 (19), 3818-3827.

9. Donth, E. The Size of Cooperatively Rearranging Regions at the Glass Transition. J. Non-Cryst. Solids 1982, 53, 325−330.
